# The roles of financial literacy and overconfidence in investment decisions in Saudi Arabia

**DOI:** 10.3389/fpsyg.2022.1005075

**Published:** 2022-09-30

**Authors:** Abdullah Hamoud Ali Seraj, Elham Alzain, Ali Saleh Alshebami

**Affiliations:** ^1^The Saudi Investment Bank Chair for Investment Awareness Studies, The Deanship of Scientific Research, The Vice Presidency for Graduate Studies and Scientific Research, King Faisal University, Al Ahsa, Saudi Arabia; ^2^College of Business Administration, King Faisal University, Al Ahsa, Saudi Arabia; ^3^Applied College in Abqaiq, King Faisal University, Al-Ahsa, Saudi Arabia

**Keywords:** financial literacy, savings, bias, Saudi Arabia, investments

## Abstract

Financial literacy has gained much attention amongst scholars, policymakers and other stakeholders due to its role in backing up investment decisions, improving personal financial management and increasing financial wellbeing. This study examines the influence of financial literacy on investment decisions with the moderating effect of the overconfidence behavioural bias. Data were collected from 180 respondents in Saudi Arabia using a questionnaire, and a convenience sampling technique was applied. The study’s findings were analysed using the partial least squares structural equation modelling (PLS-SEM) technique. It was found that financial literacy positively and significantly influenced investment decisions. Moreover, the results show that overconfidence positively affected investment decisions and that the relationship between financial literacy and investment decisions was positively and significantly moderated by overconfidence.

## Introduction

Financial literacy or awareness is necessary for choosing the right project in today’s economy, especially in developing countries. Individuals must acquire financial skills and knowledge to improve their ability to make rational investment decisions (Alshebami and Aldhyani, 2022). Due to its importance, financial literacy has attracted the attention of different groups in recent years, including financial markets, employers, bankers and governments. The importance of improving financial literacy has been emphasised due to multiple factors, such as changes in economic and demographic situations, complicated financial markets and the development of new financial markets (Al-Tamimi et al., 2009). Financial literacy refers to an individual’s ability to utilise skills and knowledge to assess and manage financial resources efficiently in the long term, with an objective of financial wellness (Abdeldayem, 2016). According to the OECD (2011), it can also be explained as the combination of investors’ understanding of financial concepts and products and their ability and skill to analyse financial opportunities and risks in making the best financial decisions and taking suitable actions to improve their financial wellness.

Financial literacy also refers to the ability to comprehend the function of money and its management and investment. It is the accumulation of skills and knowledge that enables individuals to make rational, effective and suitable decisions with their available financial resources. Financial inclusion and financial literacy are considered twin phenomena. Financial literacy is related to demand and consumers’ knowledge of demand. Financial inclusion refers to the availability and provision of financial services that meet individuals’ needs (Abdeldayem, 2016).

Financial literacy helps individuals prepare during crises and challenging times by allowing them to understand techniques and strategies to mitigate risk. It can also enforce behaviours like avoiding debt and ensuring the early payment of bills, which helps consumers maintain their accessibility to loans in the credit markets, where conditions are tight. Financially literate people comprehend the main concepts of assets and money; they are educated, knowledgeable and informed about issues related to assets, taxes, insurance, banking, investments and money management (Alshebami, 2021). They understand and plan financial decisions using their skills and knowledge.

Investment decision-making refers to the art of handling difficult situations while investing. In this cognitive process, individuals choose the most suitable options of all alternative scenarios. People cannot make investment decisions based on their available resources. Thus, the complex part of investment decision-making is carefully selecting what type of investment is most appropriate. Suitable investment decisions should be analysed considerably (Raut, 2020). Investment decisions should be made considering the rate of return, risk tolerance and various market situations. Investors’ responses to different types of market information are explained by behavioural finance. Not all investors make rational decisions, and sometimes they make biased financial decisions. Behavioural finance indicates the importance of investors’ behaviours, leading to different market anomalies (Yusnita et al., 2022). Various factors could affect the investment decisions of both individuals and corporations, including financial literacy (Ayaa et al., 2022). Investment decisions are also affected by behavioural biases, such as overconfidence (Breuer et al., 2014).

Financial crises have been faced globally during the COVID-19 pandemic, and such crises always increase costs and decrease credit accessibility in developing countries. This has also occurred in the economy of Saudi Arabia, Which is a developing country with a nascent financial sector. However, financial crises play an efficient role in developing economic behaviour by forcing people to change their financial behaviour. Amongst Arab countries, Saudi Arabia is considered to have low financial literacy, with about 29% of female and 34% of men being financially literate (Ali et al., 2021). This issue of low financial literacy and awareness persists despite major reforms and changes in the economy. People have low saving behaviour in Saudi Arabia, due to which the investment rate is also low. Individuals under the age of 35 have a particularly low tendency to invest, and most students in Saudi Arabia do not have enough knowledge about investment. This shows that people in Saudi Arabia lack financial literacy. There is, however, minimal literature globally and in Saudi Arabia about financial literacy, investment decisions and behavioural biases such as overconfidence. A study by Mian (2014) demonstrated that women were less literate than men in Saudi Arabia and that older people enjoyed higher financial literacy than younger people. Bindabel and Salim (2021) found positive relationships between investment, saving and orientation towards finance amongst Saudi university employees.

Alshebami and Marri (2022) confirmed that saving behaviour could mediate financial literacy and entrepreneurial intention amongst potential Saudi entrepreneurs, namely, students demonstrating the importance of saving behaviour as a component of financial literacy in investment orientation. In another study by Saber (2020), it was reported that developing financial literacy is critical in Saudi Arabia because of the various radical changes made by the government in relation to the economy as a result of the global reduction in oil prices, which has negatively affected the Saudi budget. The economic changes taken by the Saudi government led to a decline in individuals’ incentives, an increase in taxes imposed and an increase in some expenses, such as electricity. This forced Saudi people to adjust their financial behaviour and their spending in response to those changes. A study by Houriyah Alnakhli (2021) confirmed the absence of investment awareness amongst females in the stock market in Saudi Arabia. Accordingly, this study selected Saudi Arabia for investigating these factors related to financial literacy.

It has been reported that about 45% of people in Saudi Arabia do not have savings plans, and more than 80% do not have investment plans (Camarate and Adra, 2019). People take out loans from banks at high interest rates. Outstanding loans taken from banks for Saudi individuals amount to about US $100 in Saudi Arabia, and this figure does not include loans for housing, education and healthcare (Camarate and Adra, 2019).

These findings highlight a financial literacy gap in Saudi Arabia that should be bridged through efforts made by different stakeholders in the country to ensure high confidence and financial literacy amongst its people (LeBaron-Black et al., 2022). It is necessary to develop effective strategies for increasing financial literacy and directing individuals’ behaviour towards savings and investments. The Saudi government continues working to develop and increase financial literacy by taking suitable steps, such as arranging seminars and introducing educational courses and programmes to increase financial education (Putri and Wijaya, 2020).

The Saudi government has introduced the “Saudi Vision 2030,” in which comprehensive and effective economic reforms are proposed to increase the investment and savings of households in the country (Anastasia and Basana, 2021; Elnadi and Gheith, 2021). This vision aims to give more independence to individuals by offering them retirement plans, savings portfolios and mortgage plans. Therefore, examining the concept of financial literacy is important for improving the overall financial sector of Saudi Arabia.

Developing financial literacy means more than financial training and education and development programmes. It also emphasises some forms of social support. There can be a number of biases in investment decisions; thus, it is important to have such knowledge to avoid financial losses (Alshebami and Aldhyani, 2022). When a survey was conducted to identify which behavioural biases primarily impacted financial decisions, the results showed that those factors included availability bias, representative bias, anchoring and adjustment bias and overconfidence bias (Dangol and Manandhar, 2020).

In this study, overconfidence was examined as a moderating variable that can strengthen investment decisions. Studies have been conducted on the association between financial literacy and investment decisions, but few research studies have been conducted on the association in developing countries. This study aimed to fill this gap by focusing on Saudi Arabia. Another reason for the novelty of this study is that few studies have considered behavioural biases, such as overconfidence, in the association between financial literacy and investment decisions. Thus, this study fills this theoretical gap by responding to the call to investigate the moderating effect of overconfidence on other aspects of financial behaviour (Khan, 2016). Finally, the main goal of this research study is to examine the influence of financial literacy on investment decisions through the moderating role of overconfidence. This study provides several practical implications for policymakers involved in increasing financial literacy, with suggestions for changing financial syllabi and introducing financial management courses.

This article is organised as follows. It begins with the introduction, continues with a literature review and hypothesis development, describes the materials and methods and then provides a discussion, elaborating on the study’s contribution. It ends with a conclusion and recommendations for future study.

## Literature review and hypothesis development

### Financial literacy and investment decisions

Financial literacy refers to an individual’s ability to analyse and manage personal finances (Yusnita et al., 2022). Many researchers have investigated financial literacy from multiple perspectives, and numerous studies have been conducted to analyse investors’ financial literacy. Private organisations and governments in developed countries have conducted surveys to examine levels of financial literacy. Past studies have shown a direct association between financial literacy and investment decisions (Hilgert et al., 2003). Financial literacy can be explained as the ability to forecast effective decisions about money or financial resources and affects whether individuals use their money appropriately. Financial education programmes can improve individuals’ financial decision-making and savings (Lusardi, 2008).

Uncertainty is involved when investing money, and financial literacy helps people make safe investments. Financial literacy can save individuals from huge losses in highly turbulent and uncertain markets and can solve a number of problems. When an individual has more financial knowledge, they are more likely to utilise their finances in suitable ways. Thus, financial literacy includes an individual’s knowledge of concepts and how to apply those concepts effectively when making investment decisions (Alshebami and Aldhyani, 2022). People with lower financial literacy face more problems managing their finances and income (Delafrooz and Paim, 2011). One study showed that young Malaysian students could not save money for investments from their educational loans because they did not have financial literacy (Mohamad et al., 2008). The study showed that financial literacy positively influences individuals’ investment decisions.

People with higher financial literacy can engage in better financial behaviours and investment decisions, such as retirement plans and savings, whereas people with lower financial literacy make poor investment decisions, which negatively influence their finances (Gilenko and Chernova, 2021). Many studies have examined the impact of financial literacy on investment decisions, but few have investigated the association between financial literacy and saving behaviour that leads to investment decisions in developing countries like Saudi Arabia (Supanantaroek et al., 2017). According to Alshebami and Aldhyani (2022), when a high level of financial literacy is developed, it helps individuals make rational investment decisions.

Lusardi and Olivia (2007) examined the financial literacy of 12 countries, including Australia, Japan, the EU, the United States and the United Kingdom, and found that overall, respondents had low financial literacy (Lusardi and Olivia, 2007). Financial literacy has significant and far-reaching implications for investment behaviour and savings. The basic rule of thumb dominates saving behaviour in a household lacking basic financial information. Those who have received financial education save more than those without such knowledge. People with low financial literacy are less likely to have retirement plans and fail to save enough money (Supanantaroek et al., 2017).

In the US, Few US residents are sure about the sufficiency of their retirement plans, but limited research is available regarding why people fail to have them. Therefore, a module in this study was developed based on financial literacy that was used to reveal how people make decisions and gather information when making investment decisions. The findings revealed that older US residents had financial literacy, and only half of the older residents knew about inflation and compound interest. Further, about one-third of the US residents knew about risk diversification. And those individuals without college degrees, minorities and women had the lowest levels of financial literacy. People with financial literacy understand savings and investing in bonds and stocks, termed complex assets (Lusardi and Olivia, 2007).

A question was raised regarding the lower demand for financial literacy in developing or emerging markets. One school of thought argues that limited financial literacy and cognitive ability affect demand. The second school of thought posits that the reason for lower demand is expensive financial products (Miłaszewicz, 2019). The research study included a field experiment and questionnaires that aimed to differentiate between the two views. A survey from Indonesia and India showed that financial literacy is a significant demand predictor for financial products. The study further revealed that financial literacy is an essential factor for the individuals’ well-being, development of the economy, investments, savings and sustainability (Haleem et al., 2022). Based on previous studies, the following hypothesis is postulated:

*H1*: Financial literacy positively influences investment decisions.

### Overconfidence and investment decisions

According to financial market theory, the behavioural finance approach examines the effect of psychology on the stock market and investors’ behaviours. This theory discusses how individuals behave rationally with specific constraints. The behavioural finance approach integrates financial and classical economic theories of decision-making and psychology (Miłaszewicz, 2019). Anomalies of the efficient market hypothesis indicate how individuals’ behaviours can be described and explained in behavioural finance (Gill and Bajwa, 2018). Costa et al. (2019) stated that the main objective of behavioural finance is to comprehend how individuals make investment decisions. However, investors can make mistakes when making these decisions, and overconfidence is one of these mistakes (Ayaa et al., 2022).

According to Pikulina et al. (2017), overconfidence is a common and well-established bias that leads individuals to ignore the risks associated with investment and become too confident about their skills and knowledge. Overconfidence, an overly positive self-assessment of ability, has gained the attention of many researchers in the financial sector. Overconfidence makes investors feel smarter and more knowledgeable so that when they predict an event, they may perceive it as certain. According to Pompian (2012), reality is sometimes contrary to expectations. Overconfidence is an individual’s overestimation of their own performance, abilities and chances of success. It is a belief that they have better understanding and judgement than other people.

Overconfident people override the evidence available in a situation because they are rigid and certain about their beliefs. They are so sure about their views that they ignore important information when making investment decisions (Eichholtz and Yönder, 2015). Past studies have examined the influence of overconfidence on investment decision-making. When investors with discount brokerage accounts are involved in excessive trading and become overconfident, they are known to trade more frequently, which may result in negative returns (Ayaa et al., 2022). Pikulina et al. (2017) found that the behavioural bias of overconfidence leads to extreme investment decisions. Overconfident investors expect low risks and high returns when investing, but this is not guaranteed. Palomino and Sadrieh (2011) and Chu et al. (2012) noted that overconfident investors engaged in stock transactions that negatively affected returns. McCannon et al. (2016) found that overconfident investors made risky investment decisions. According to Breuer et al. (2014), people with high overconfidence are more courageous when allocating funds to high-risk assets, but the actual results are not always favourable. Overconfident investors take insufficient time for profit realisation and retain stocks that have suffered losses. Eichholtz and Yönder (2015) noted that overconfidence leads to high-risk investment decisions. Therefore, the following hypothesis was formulated based on the empirical evidence:

*H2*: Overconfidence increases the likelihood of investment decisions.

### Moderating role of overconfidence

Plous (1993) developed the psychology of judgement and stated that decision-making means selecting between many alternatives. Therefore, for an individual to make a good decision, they should have solid judgment. Accordingly, decision-makers who are overconfident have inflated certainty’ about their skills. Thus, they undervalue alternative options and ignore new information when making decisions. The behavioural bias of overconfidence is highest in complex tasks and amongst experts. Psychologists call this pattern self-enhancing bias, which complements confirmation and heuristic bias (Pikulina et al., 2017). Overconfidence leads to decision-makers overestimating their knowledge (Chu et al., 2012), so they are surprised by sudden events and underestimate the unpredictability of those events. Overconfidence is a behavioural bias that sometimes leads to incorrect investment decisions. It is generally understood that overconfident individuals have only half of the correct knowledge and information about investment decisions. Overconfident individuals underestimate their chances of loss, leading to excessive trading and high-risk portfolios (Dangol and Manandhar, 2020).

However, overconfidence does not always need to generate wrong financial decisions. Wan et al. (2022) argued that overconfidence encourages risk-prone investment, which can lead to higher returns. An aggressive and overconfident trader may achieve more benefits than a slow-moving trader. This situation is an inverse arbitrage when irrational decision makers thrive at the expense of rational decision makers (Eichholtz and Yönder, 2015).

According to McCannon et al. (2016), overconfidence is the difference between an individual’s beliefs about their financial knowledge and their actual competence. Overconfidence can be measured in two ways. One way is overestimation, which refers to self-beliefs about control, ability or achievements that are higher than the actual results. The other is overplacement, which refers to an individual’s perception of themselves as being better than others. Overplacement and overestimation lead to overestimated performance in comparison with others’ performance or actual performance. Individuals make investment decisions in various investment projects when their income depends on financial literacy and risk.

A study conducted on students with economics degrees and investment experience revealed that confidence amongst the students was higher than their actual knowledge, and most students believed that they were better than others in relation to financial literacy. Professional managers and investors have more knowledge than students, but they are still overconfident in their investment decisions (Wan et al., 2022). When financially literate individuals are overconfident, their investment decisions are impacted. Overconfidence, one of the most important behavioural biases, moderates the association between financial literacy and investment decisions. Wan et al. (2022) also found that overconfidence is a moderator in investment decisions. Pikulina et al. (2017) noted that overconfidence makes financially literate investors more enthusiastic about investments, leading to risks being taken to generate high returns. Hence, based on the discussion, it is hypothesised that:

*H3*: Overconfidence positively moderates the relationship between financial literacy and investment decisions.

### Conceptual model

**Figure 1:**
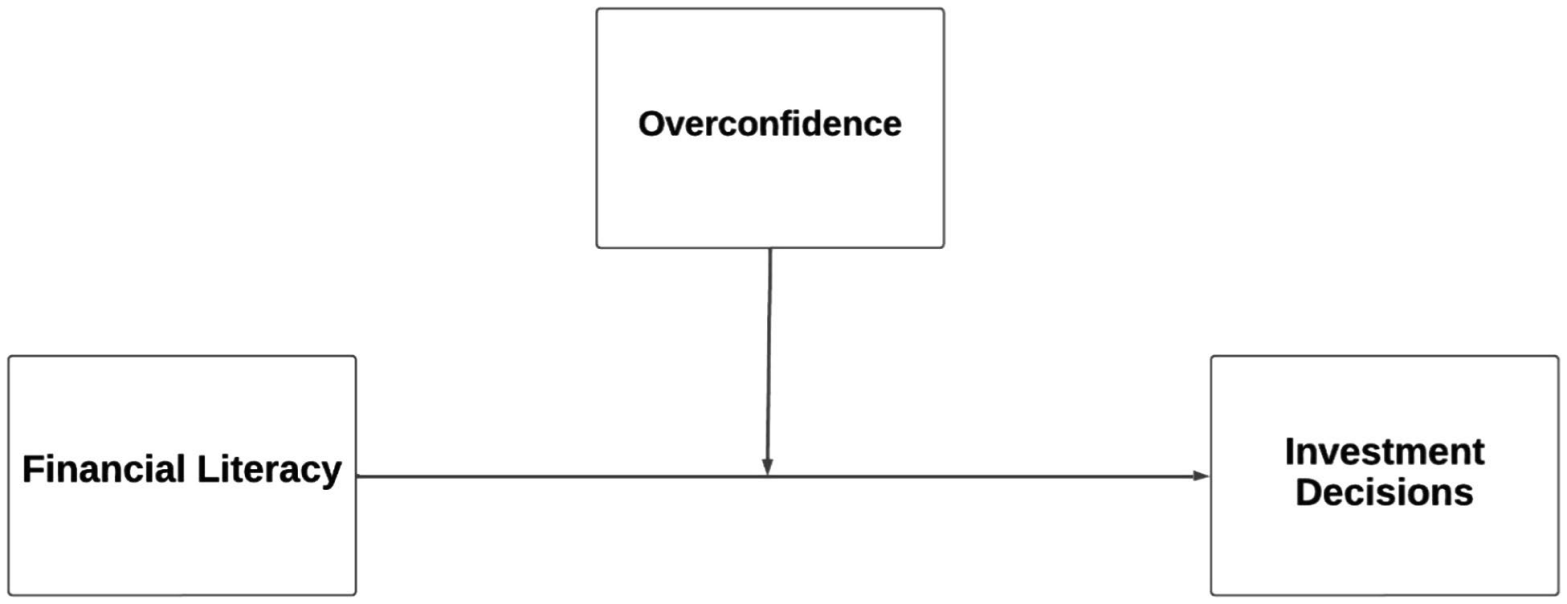
Structural Model. Source: Authors’ elaboration.

The conceptual model depicting the relationships and hypotheses is shown in Figure 1. Financial literacy is the independent variable and investment decisions is the dependent variable. Overconfidence is the moderator variable in this study.

## Methodology

### Sample and data collection

The nature of this study was cross-sectional and based on quantitative data. The sample was collected from individuals from the general public in Saudi Arabia from April to June 2022. The financial literacy rate, saving rate and investment decisions in Saudi Arabia are low, particularly amongst young individuals who fear starting a new business (Hasler and Lusardi, 2017; The General Authority for Statistics, 2020). To address this issue, a sample of 180 respondents (123 male and 57 female) was selected, and the proposed model was examined to understand how financial literacy and overconfidence could push the participants towards investments, particularly with the support of the Saudi Vision 2030. The convenience sampling technique was utilised because of its ease of reaching survey respondents and its low requirements of time and money (Zikmund, 1997). A pilot test with 20 respondents was conducted to evaluate the suitability of the self-administered questionnaire. The results of the pilot test were satisfactory. The original draft of the questionnaire was in the English language, and translation was checked using the back-translation process by two language experts with a good command of English and Arabic. No incentive was offered to the respondents. We assured the participants that their responses were confidential and were only used for academic research purposes.

### Measures

The items measuring the constructs were adapted from existing measures used by prior researchers to assess the influence of financial literacy, overconfidence and investment decisions. All measurement items were anchored on a 5-point Likert scale ranging from ‘strongly disagree’ to ‘strongly agree’.

#### Financial literacy

We used seven items from Khan’s (2016) previous study to measure financial literacy. One example of an item is, ‘I am somewhat knowledgeable of stock market activities that happen in the market’. The composite reliability for financial literacy was 0.906.

#### Overconfidence

Overconfidence is one dimension of behavioural bias. To assess overconfidence, we used five measurement constructs from a prior study by Lin (2011). One sample item is, ‘I am sure I can make the correct investment decisions’. The composite reliability for overconfidence was 0.881.

#### Investment decisions

To measure investment decisions, we used a five-item scale adopted from Khan’s (2016) study. A sample item is, ‘It is more satisfying to save than to invest money’. The composite reliability for investment decisions was 0.762.

### Common method bias

As the study data are based on self-reports, to ensure adequate scale reliability and convergent validity and instil confidence in the study findings, preventive procedural and statistical measures were taken to reduce possible method variance. The scale items were randomly mixed in the survey during data collection. We also checked for common method variance during the data analyses. Harman’s single-factor test was first conducted by entering all the key variables into an exploratory factor analysis (EFA), and the results showed that no single factor emerged. Moreover, the ‘forced’ single-factor solution accounted for 32.47% of the variance, which is lower than the recommended 50% threshold (Podsakoff et al., 2003), indicating that CMV was not a major concern in this study.

## Results

### Data analysis method

To test the proposed model, we adopted partial least squares structural equation modelling (PLS-SEM) using Smart PLS software (version 3.3.8) due to our exploratory purposes and the small number of constructs and indicators (Hair et al., 2011, 2012). The analysis process included a measurement model and a structural model.

### Measurement model

There are two kinds of constructs: formative and reflective (Qalati et al., 2022b). Based on the definition of reflective constructs, the items of one reflective construct should be based on the same or similar content (Hair et al., 2013). Accordingly, all constructs were treated as reflective. We followed the guidelines for evaluating reflective constructs, consisting of internal reliability, indicator reliability, convergent validity and discriminant validity (Henseler et al., 2014). As Table 1 shows, the values of all constructs’ composite reliability (CR) were greater than 0.70 (Henseler et al., 2014; Hair et al., 2019), indicating that all constructs had good internal reliability. Composite reliability is assumed to be better than other internal reliability measures, such as Cronbach’s alpha, as it produces more values. Indicator loadings of ID3, ID5 and ID6 less than 0.60 were eliminated from the measurement model (Chin, 1998), and nearly all indicators indicated good reliability. The other indicator loadings had values above 0.60, which is acceptable according to Fang et al. (2021), Nunnally (1978), and Stevens (1992).

**Table 1:** Measurement model.

Constructs	Loadings	Composite reliability	Average variance extracted (AVE)
Financial Literacy		0.906	0.581
FL 1	0.725		
FL 2	0.837		
FL 3	0.851		
FL 4	0.703		
FL 5	0.788		
FL 6	0.779		
FL 7	0.631		
Overconfidence		0.881	0.599
OC 1	0.811		
OC 2	0.866		
OC 3	0.762		
OC 4	0.790		
OC 5	0.621		
Investment Decisions		0.762	0.445
ID 1	0.627		
ID 2	0.684		
ID 4	0.669		
ID 7	0.686		

Source: Primary data.

The average variance extracted (AVE) of financial literacy and overconfidence construct values were larger than 0.50, indicating that these constructs possess a high degree of convergent validity (Hair et al., 2012). The investment decisions construct had an AVE of less than 0.50. Table 2 shows that the square root of each construct’s AVE was greater than the correlation of the construct to other latent variables (Chin, 1998), and Table 3 shows that the Heterotrait–Monotrait Ratio (HTMT) values were all smaller than 0.85 (Hair et al., 2011). These three tables indicate that all constructs possessed a high degree of convergent validity.

**Table 2:** Fornell–Larcker criteria.

	FL	FL*OC-ID	ID	OC
FL	0.762			
FL*OC-ID	0.002	1.000		
ID	0.600	0.588	0.667	
OC	0.608	0.835	0.656	0.774

Source: Primary data.

**Table 3:** HTMT ratio.

	FL	FL*OC-ID	ID	OC
FL				
FL*OC-ID	0.095			
ID	0.801	0.212		
OC	0.712	0.126	0.909	

Source: Primary data.

### Assessing the structural model

Before evaluating the structural model, we tested the variance inflation factor (VIF) to assess the presence of collinearity amongst the indicators of the study constructs. If VIF values are above 5, this indicates the presence of collinearity amongst the predictor constructs (Qalati et al., 2022a). Ideally, the VIF values should be around 3 or less (Hair et al., 2019). In our study, all the values of the predictor constructs were less than 3 (FL = 1.595, OC = 1.609, FL* OC-ID = 1.015), confirming the absence of any collinearity.

**Figure 2:**
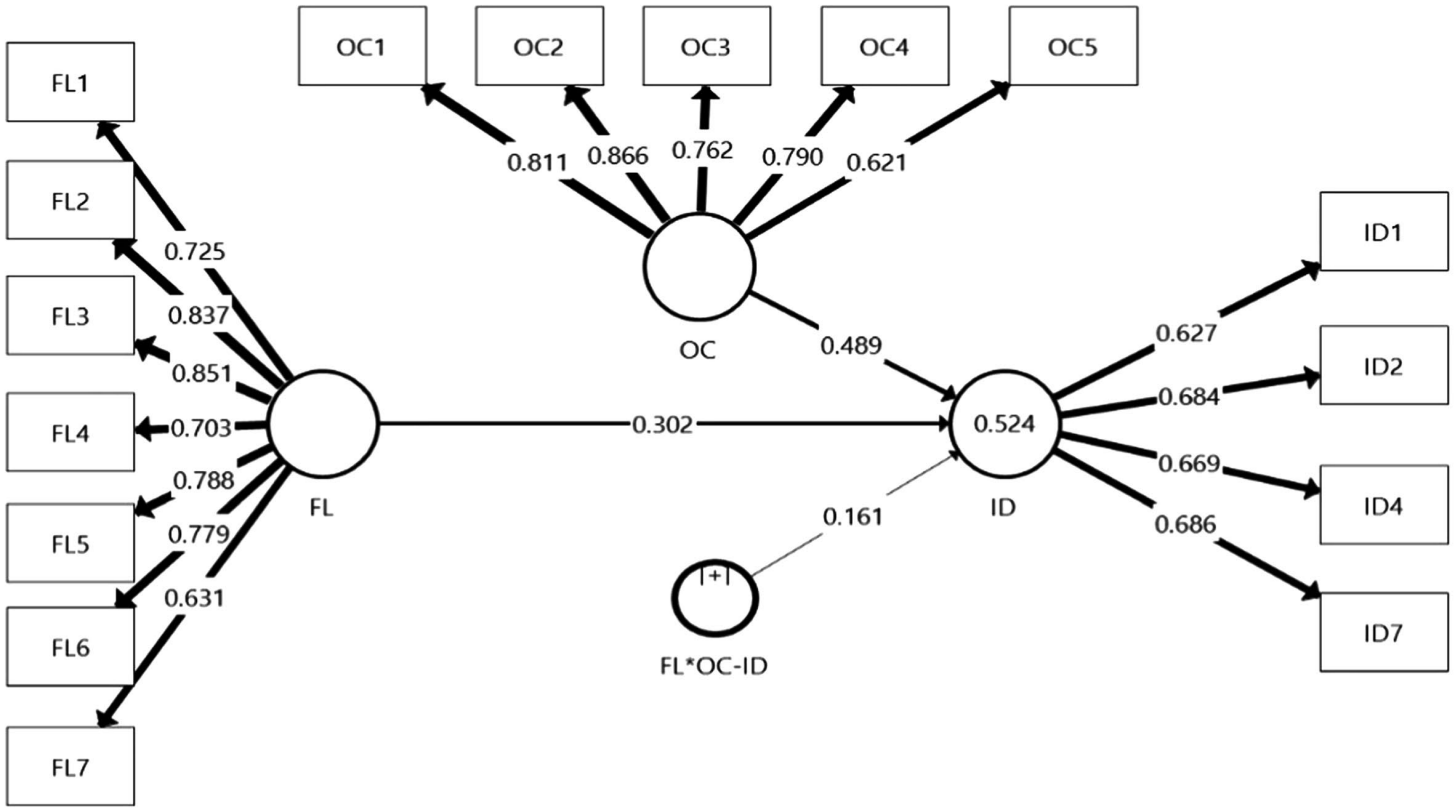
Structural Model. Source: Primary data.

We then continued to follow the rules of evaluating the structural model (Hair et al., 2013). The results of the structural model are shown in Figure 2. The coefficient of determination (Ali et al., 2021) indicates the number of independent variables explained by the dependent variables (Chin, 1998). R^2^ values were calculated for all constructs with values of 0.190, 0.333 and 0.670, suggesting that endogenous latent variables in the structural model were weak, moderate and substantial, respectively (Hair et al., 2012). The value of R^2^ = 0.524 for investment decision was considered moderate. To assess the path coefficients’ significance in structural path analysis, the signs and statistical significance of the path coefficients were used to test the proposed hypotheses through 5,000 bootstrap samples (Hair et al., 2011), and the path coefficients needed to be significant at the 0.05 level (*t*-value >1.96).

### PLS predict

The PLSpredict algorithm is implemented in the software of the SmartPLS. This PLSpredicte algorithm permits researchers to get prediction error summaries, the k-fold cross-validated prediction errors and statistics such as the mean absolute error (MAE), the root mean square error (RMSE) and the mean absolute percentage error (MAPE) to evaluate the predictive performance of their PLS path model for the latent variables and manifest variables. The availability of the PLSpredict algorithm helps the authors to compare the predictive performance of available different PLS path models. Both mean absolute deviation MAE and the RMSE are used when we aim to select the predictive model amongst different models. Researchers must choose a suitable model that reduces RMSE and MAD values in the latent variable scores after comparing the values of the MAD and RMSE (Shmueli et al., 2016).

To understand the results of a specific PLS path model, we have to check them and compare them with the benchmarks provided, i.e. the Q^2^ value and the linear regression model (LM). If the Q^2^ value shows a positive finding, it indicates that the prediction error of the PLS-SEM results is smaller than the prediction error of simply using the mean values. In that case, the PLS-SEM models offer better predictive performance. Comparing the PLS-SEM results provides information on whether using a theoretically established path model improves the predictive performance of the available indicator data. Furthermore, the second step is to compare the result of the LM outcomes with the PLS-SEM results. The PLS-SEM should have a lower prediction error (e.g. in terms of RMSE or MAE) than the LM (Shmueli et al., 2016).

According to the findings in Table 4, the indicators in the PLS-SEM had lower values than the linear model (LM) benchmark, indicating the high prediction power of the PLS-SEM. Furthermore, we found a positive value for Q2, confirming that the prediction error of the PLS-SEM results is smaller than the prediction error of the LM, demonstrating PLS-SEM’s superior predictive ability.

**Table 4:** MV prediction summary.

PLS				
	RMSE	MAE	MAPE	Q^2^_predict
ID2	1.033	0.827	35.251	0.234
ID4	0.996	0.814	37.773	0.259
ID1	1.038	0.829	31.143	0.106
ID7	0.736	0.570	17.757	0.246
LM				
	RMSE	MAE	MAPE	Q^2^_predict
ID2	1.067	0.861	35.930	0.183
ID4	1.018	0.804	35.597	0.227
ID1	1.090	0.869	32.310	0.015
ID7	0.736	0.558	17.226	0.246

Source: Primary data.

### Hypothesis testing

We tested the proposed hypotheses, and the results are shown in Table 5. The H1 results show that financial literacy was positively and significantly related to investment decisions (*β* = 0.302; *t* = 3.748; *p* < 0.001), and H1 was supported. H2 findings indicate that overconfidence was positively and significantly related to investment decisions (*β* = 0.489; *t* = 6.525; *p* < 0.001). Therefore, H2 was accepted. H3 results reveal that overconfidence positively and significantly moderated the relationship between financial literacy and investment decisions (*β* = 0.161; *t* = 3.392; *p* < 0.001), so H3 was supported. Additionally, the value of the predictive relevance (Ali et al., 2021) of investment decisions (Q^2^ = 0.213), larger than zero, indicates the model’s predictive relevance for latent variables (Chin, 1998). Finally, Cohen’s (1988) effect size (Ali et al., 2021) showed the effects of latent independent variables on latent dependent variables larger than 0.020, 0.150 and 0.350, indicating low, medium and large effects, respectively (Hair et al., 2012). The effect size of financial literacy on investment decisions was medium, and the overconfidence effect on investment decisions was large, attaining values between 0.120 and 0.313.

**Table 5:** Structural model.

Hypotheses	(R^2^ = 0.524 and Q^2^ = 0.213)	β	t	p
Direct Relationships				
H1	Financial Literacy→Investment Decisions	0.302	3.748	0.000
H2	Overconfidence→Investment Decisions	0.489	6.525	0.000
Moderating Relationships				
H3	Financial Literacy*Overconfidence→Investment Decisions	0.161	3.392	0.001

Source: Primary data.

### Moderation analysis

**Figure 3:**
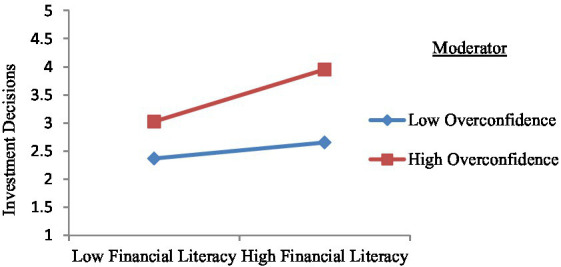
Moderation Slope. Source: Primary data.

Figure 3 shows the moderation analysis of the overconfidence variable on the relationship between financial literacy and investment decisions. The figure shows that the higher an individual’s overconfidence and financial literacy, the more likely the investment decisions can occur. This confirms H3.

In conclusion, overconfidence strengthens the positive relationship between financial literacy and investment decisions.

## Discussion

All three hypotheses of this study were supported. It was found that financial literacy was significant for improving the rationality of investment decisions of individuals (Al-Tamimi et al., 2009; Awais et al., 2016; Ansari et al., 2022). Thus, it is important to examine the major factors affecting financial literacy amongst the people of Saudi Arabia. We also examined how greater financial literacy leads to positive investment decisions, as moderated by overconfidence. First, we investigated the impact of financial literacy on investment decisions in the first hypothesis. This association was in line with previous research stating that people with financial literacy could invest their finances in the right projects (Supanantaroek et al., 2017).

People with lower financial literacy make irrational investment decisions because they have insufficient knowledge to manage their finances. People know which factors to consider when making investment decisions when they have financial knowledge. Thus, they succeed in achieving high returns after making rational investments. Gilenko and Chernova (2021) noted that people with knowledge of financial management make investment decisions about saving plans effectively. Supanantaroek et al. (2017) also confirmed the influence of financial literacy in improving savings attitude and behaviour, which could later lead to good investments. These findings also align with Khan’s (2016) results, which confirmed the positive relationship between financial literacy and investment decisions. Abdeldayem (2016) revealed that participants with low financial literacy avoid investing in complex financial products with high risk and higher returns, confirming the need for a high level of financial literacy to ensure better investment. The findings of our first hypothesis indicate consistency with these previous studies.

The second hypothesis examines the impact of overconfidence on investment decisions. Previous researchers have claimed that investors are ready to take risks by investing funds in more options when they have enough confidence. Therefore, high confidence increases rational investment decisions (McCannon et al., 2016). This study examines the impact of overconfidence on investment decisions using the behavioural finance approach. Ayaa et al. (2022) revealed that overconfidence is a behavioural bias but that it positively affects investment decisions. Pikulina et al. (2017) reported that when investors are overconfident, they become extremists in investing. As a result, they generate a high rate of returns because they take high risks. Other studies have also confirmed the influence of overconfidence on investment decisions (Jain et al., 2020; Rashid et al., 2022). Chu et al. (2012) claimed that when investors are overconfident, they want to invest more money, and Breuer et al. (2014) also shed light on the positive association between overconfidence and investment decisions because such investors are courageous in taking risks and investing. McCannon et al. (2016) noted that overconfident investors are risk takers, so they do not hesitate to make many investment decisions. Therefore, the findings of our second hypothesis are consistent with those of previous studies.

The third hypothesis investigates the moderating impact of overconfidence on financial literacy and investment decisions. Pikulina et al. (2017) argued that people with financial literacy make the right investment decisions, and when they are overconfident when investing, this association becomes stronger. They also found that overconfidence strengthens the relationship between financial literacy and investment decisions. Likewise, Ayaa et al. (2022) noted that overconfidence strengthens the association between financial literacy and investment decisions. They noted that by using the behavioural finance approach, investors take more risks to obtain high returns because of their overconfidence. However, high returns are not always guaranteed. Sometimes, they get lower returns, but due to their overconfidence, they invest in multiple projects. Thus, the results of our third hypothesis are consistent with previous researchers’ arguments, stating that overconfidence strengthens the relationship between financial literacy and investment decisions.

## Theoretical and practical implications

### Theoretical implications

The current study is one of the few studies investigating the relationship between financial literacy and investment decisions through the moderating role of overconfidence in Saudi Arabia using the behavioural finance approach. This study posits a theoretical model that is beneficial to investors when making investment decisions. It contributes to the existing literature and to the body of knowledge on the role of financial literacy in making good investment decisions. The study also shows the moderating role of overconfidence, indicating that when financially literate investors are overconfident, they tend to make better investment decisions (Ayaa et al., 2022). This study also provides recommendations and guidelines for investors and stakeholders for improving financial literacy amongst Saudi Arabian people to help them make profitable investment decisions for better financial position and higher returns.

### Practical implications

Investors sometimes act irrationally when making investment decisions. Several factors affect their investment decisions and lead them to display cognitive and behavioural biases that facilitate deviations from rational behaviour, such as overconfidence. Financial literacy amongst individuals can be developed by introducing financial education through revisions of courses and syllabi. Financial education can provide comprehensive financial consultation and training to individuals and workshops could be offered by the government and other organisations in the country. This objective can be supported by calling financially literate peers and parents to take part in the movement to increase public financial literacy. The Saudi government should help individuals develop a good understanding of financial literacy by enforcing regulations that encourage and assist residents to learn more about investment decisions. Moreover, the Saudi government should work with commercial banks and other financial institutions to develop essential financial services and products, financial programmes and financial training for people to ensure rational investment decisions.

This study focuses on increasing confidence amongst individuals to improve benefits and help them learn to make rational investment decisions by utilising their financial literacy. The current study highlights the importance of spreading awareness about financial literacy to benefit Saudi Arabia because of the low rate of financial literacy in the country. Finally, this study provides practitioners and researchers with valuable insights into how financial literacy impacts investment decisions and which factors can strengthen this association (i.e. overconfidence).

### Limitations and future research directions

This study has some limitations that future researchers can address. It was limited to a small sample size, and a larger sample size could provide more generalisable results in future studies (Bougie and Sekaran, 2019). The other limitation is that this study collected data using questionnaires. Investors and professionals from Saudi Arabia could be interviewed in the future to obtain more comprehensive information on financial literacy and investment decisions. This study is based on quantitative data, but qualitative data could provide valuable insights into the opinions and thoughts of investors to achieve a better interpretation and understanding of the association between financial literacy and investment decisions in Saudi Arabia.

This study examined the moderating role of overconfidence. Future researchers could examine the association between financial literacy and investment decisions through the moderating role of demographic factors, such as education, gender, monthly income, age, employment status or workplace activity. Future studies could also add some mediating variables to the association between financial literacy and investment decisions. The current study is based on cross-sectional data, and in the future, researchers could focus on a longitudinal study to investigate the causal impact of financial literacy on investment decisions. Finally, this study used convenience sampling, and simple random sampling or other sampling methods could be used in the future to ensure minimal bias in the data (Bougie and Sekaran, 2019).

## Conclusion

The major goal of this study was to investigate the impact of financial literacy on investment decisions through the moderating role of overconfidence using the behavioural finance approach. All hypotheses were accepted and were consistent with previous research. Poor financial literacy prevails globally, especially in developing countries, including Saudi Arabia. This indicates the importance of investigating the factors affecting the development of financial literacy and investment decisions and the extent to which overconfidence can strengthen this association. This study was conducted to examine the condition of financial literacy amongst the people of Saudi Arabia. The findings revealed that financially literate investors make good investment decisions, and when they are overconfident, this association is strengthened. Thus, financial literacy is important for enabling people to make rational investment decisions so that they can effectively manage their finances. Individuals or investors who lack financial literacy make irrational investment decisions, leading to financial losses.

This study also shows how investors’ overconfidence in their skills and abilities impacts their investment decisions. Underconfident people make irrational investment decisions and do not want to take risks. Thus, a moderate level of overconfidence leads to good investment decisions. The Saudi government should take radical steps to add financial management information to educational courses so that students become familiar with the concept of financial literacy and can apply it in their practical lives in the future. Also, should work on introducing seminars, workshops and other ways to spread financial literacy in the country in cooperation with other organisations.

## Data availability statement

The raw data supporting the conclusions of this article will be made available by the authors, without undue reservation.

## Ethics statement

The study was conducted according to the guidelines of the Declaration of Helsinki and approved by the Ethics Committee of King Faisal University. The approval number was (KFU-REC-2022-MAY –ETHICS2) and the date of the approval was 24 May 2022. The patients/participants provided their written informed consent to participate in this study.

## Author contributions

All authors contributed to the article and approved the submitted version.

## Funding

This work was supported by the Saudi Investment Bank Chair for Investment Awareness Studies, the Deanship of Scientific Research, the Vice Presidency for Graduate Studies and Scientific Research, King Faisal University, Saudi Arabia [Grant No. 146].

## Conflict of interest

The authors declare that the research was conducted in the absence of any commercial or financial relationships that could be construed as a potential conflict of interest.

## Publisher’s note

All claims expressed in this article are solely those of the authors and do not necessarily represent those of their affiliated organizations, or those of the publisher, the editors and the reviewers. Any product that may be evaluated in this article, or claim that may be made by its manufacturer, is not guaranteed or endorsed by the publisher.
